# COLORECTAL CANCER: IS THERE A T OR B CELL SIGNATURE RELATED TO THE PATIENT OUTCOME?

**DOI:** 10.1590/S0004-2803.24612025-146

**Published:** 2026-08-14

**Authors:** Valquiria BUENO, Juliana do Carmo Barros SANTOS, Daniela FRASCA, Nora Manoukian FORONES

**Affiliations:** 1 Universidade Federal de São Paulo UNIFESP, Escola Paulista de Medicina, Professor Associado, São Paulo, SP, Brasil.; 2 Universidade Federal de São Paulo UNIFESP, Escola Paulista de Medicina, Graduação, São Paulo, SP, Brasil.; 3 Universidade de Miami, Miller School of Medicine, Professor Associado, Flórida, EUA.; 4 Universidade Federal de São Paulo UNIFESP, Escola Paulista de Medicina, Professor Titular, São Paulo, SP, Brasil.

**Keywords:** Colorectal cancer, T lymphocytes, B lymphocytes, disease relapse, progression-free survival, Câncer colorretal, linfócitos T, linfócitos B, recidiva da doença, sobrevida livre de progressão

## Abstract

**Background:**

Colorectal cancer (CRC) is the third more common cancer with around 1.9 million people diagnosed in 2020. Age is an important risk factor for CRC development and older patients have the lowest survival rates which has been associated with age-related comorbidities and less aggressive treatment. Older individuals present a systemic low-level of chronic inflammation (inflammageing) and, at the same time, they develop a less efficient adaptive immune response, in addition to the changes in innate immunity with an overall negative impact in the immune response (immunosenescence). These processes contribute for tumor cell evasion of the immune surveillance and development of a tumor microenvironment (TME) favorable for metastatic spread.

**Objective:**

T or B cell signature related to the patient outcome after therapy could be a very useful tool to anticipate the treatment of disease relapse or metastasis.

**Methods:**

We evaluated 24 patients with CRC in a follow-up of at least 24 months and correlated with T and B cells subsets.

**Results:**

We found that patients with disease relapse (n=2) presented the highest levels of double negative B cells (B DN). Other patients with high levels of B DN were classified as CRC 2 group and presented also lower IgM memory and higher switched memory B cells.

**Conclusion:**

Our findings suggest a possible exploratory association between alterations in B-cell subsets and clinical outcomes. A higher number of patients and further characterization of DN B cells are fundamental to use these cells as prognostic biomarker in CRC.

## HIGHLIGHTS

• There are scarcity of data about the use of T and B lymphocytes as biomarkers for the outcome of patients with colorectal cancer.

• It was performed the staining of T and B lymphocytes subsets in the peripheral blood of patients with colorectal cancer and these findings were correlated with the patients outcome.

• Two patients with disease relapse after CRC treatment presented higher percentages of B double negative lymphocytes.

## RESUMO

**Contexto:**

O cancer colorretal (CCR) é o terceiro câncer mais comum com cerca de 1.9 milhões de pessoas diagnosticadas em 2020. O envelhecimento é um importante fator de risco para o desenvolvimento de CCR e pacientes idosos têm as menores taxas de sobrevida o que tem sido associado com as comorbidades relacionadas ao envelhecimento e tratamento menos agressivo. Os indivíduos idosos apresentam inflamação crônica sistêmica de baixo nível, e ao mesmo tempo, eles desenvolvem uma resposta imune adaptativa menos eficaz, além de alterações na imunidade inata com um impacto negativo na resposta imune global (imunosenescência). Tais processos contribuem para que o microambiente tumoral (TME) seja favorável à metástase.

**Objetivo:**

Uma assinatura de células T ou B que se correlacione com a evolução do paciente após terapia poderia ser muito útil para antecipar o tratamento da recidiva do CCR ou metástase.

**Métodos:**

avaliamos 24 pacientes com CCR num seguimento de pelo menos 2 anos e correlacionamos com os subtipos de células T e B.

**Resultados:**

Pacientes com recidiva da doença (n=2) apresentaram os maiores níveis de células B duplo negativas (B DN). Outros pacientes com altos níveis de B DN foram classificados como grupo CRC 2 e apresentaram baixos níveis de células B de Memória IgM e altos níveis de células B de Memória com mudança de classe de Ig.

**Conclusão:**

os achados sugerem uma possível associação exploratória entre as alterações nos subtipos de células B e desfecho clínico. A avaliação de um número maior de pacientes e a mais detalhada caracterização das células B DN são fundamentais para o uso dessas células como biomarcadores de prognóstico em CRC.

## INTRODUCTION

Colorectal cancer (CRC) is a complex disease and accounts for 10% of all new global cases of cancer. CRC is the third more common cancer with around 1.9 million people diagnosed in 2020^1^. Age is an important non-modifiable risk factor for CRC development and the incidence of this cancer can double in each successive decade between the ages of 40 and 80 years^2^. Patients’ percentage of diagnostic at the time of metastatic spread at the age of 50-64 years is higher (23%) than at ≥65 years (19%). However, older patients have the lowest survival rates which has been associated with age-related comorbidities and less aggressive treatment^3^. Not only comorbidities impact CRC outcomes in older individuals, but also other age-related features such as premature cell senescence caused by metabolic stress, oncogenes overexpression or loss of tumor suppressor genes, and chemotherapy or radiotherapy^4^. In aged organisms senescent cells accumulate and present changes in the expression of epigenetic genes and metabolism, exhibiting a senescence-associated secretory phenotype (SASP)^5^ ^,^ ^6^ ^,^ ^7^. In terms of functional profile SASP corresponds to secretion of pro-inflammatory cytokines, chemokines, growth factors, and extra-cellular matrix degrading proteases leading to a systemic low-level of chronic inflammation (inflammageing)^6^. Thus, at the same time that senescence is linked to the blockade of primary and malignant cells proliferation, SASP can promote tumorigenesis by inducing senescence in nearby non-senescent cells and components of stroma^4^ ^,^ ^8^ ^,^ ^9^ ^,^ ^10^. In addition, ageing has been associated to a process of less efficacy of adaptive immunity and changes in innate immunity with an overall negative impact in the immune response^11^. The less efficient immune system can contribute for tumor cell evasion of the immune surveillance and development of a tumor microenvironment (TME) favorable for metastatic spread. In this scenario, few articles discuss whether there is an adaptive immune system’s signature in CRC. Zhang et al. compared CRC patients (57-72 years old) with healthy donors at similar age and observed a reduction of total T cells, helper T cells (T CD4+), cytotoxic T cells (T CD8+), and double negative T cells (CD4-CD8-) in patients. They also found an increased percentage of B cells, plasmablasts and activated T cells. The comparison between early-stage and advanced-stage patients showed decrease of CD8+ naïve T cells and increase of CD8+ and CD4+ terminally differentiated effector T cells (EMRA). These findings suggest that advanced stages of CRC are associated with a T cell phenotype that resembles immunosenescence^12^. Bueno et al. found in a metastatic CRC patient, lower percentage of total CD4+ and naïve T cells in comparison with healthy control and non-metastatic CRC patient. Nevertheless, total CD8+ T cells, naïve CD8+ T cells and EMRA CD8+ T cells were increased in CRC patients when compared with control^13^. Tada et al. showed that the pretreatment immune status correlates with the outcome of metastatic CRC patient after first-line chemotherapy. It was observed in peripheral blood that low percentages of CD4+ and CD8+ effector memory T cells (TEM) were associated with shorter progression-free survival^14^. Therefore, our hypothesis is that the percentage of T and B cells and their subsets in peripheral blood could be used as a signature for prognosis in patients with CRC. If so, a time line of blood analysis could be used to predict patient outcome after treatment (free-disease survival, relapse, metastasis) and in case of disease progression, it would be possible to provide earlier therapy. Therefore, it was our aim to evaluate, whether there is a prognostic-associated T or B cell signature in CRC patients followed-up for at least 2 years.

## METHODS

### Participants

Patients diagnosed with colorectal cancer (CRC) that undergone curative resection and/or chemotherapy/radiotherapy at Hospital São Paulo - UNIFESP - EPM were evaluated. Men (n=10) and women (n=14) >50 years of age and with a minimum follow-up of 24 months after diagnosis were included in this study. Patients provided written informed consent and the study was approved by the UNIFESP Ethics Committee (Plataforma Brasil 67406023.3.0000.5505).

### Blood Samples and Flow cytometry

Blood was collected (4 mL) in Vacutainer EDTA tubes and PBMCs were isolated by Ficoll-Hypaque gradient and centrifugation (1800 rpm at 30 minutes). PBMCs were collected, washed twice and adjusted for staining with monoclonal antibodies (20 minutes at room temperature).

Monoclonal antibodies anti-CD3 FITC, anti-CD4 PE-Cy7, anti-CD8 APC-Cy7, anti-CD27 APC, anti-CD19 PE, anti-IgD PerCP- Cy5.5, anti-IgM APC-Cy7 (BioLegend), anti-CD45RA PE (Ebiosciences) were used to evaluate T and B cells. After membrane staining, cells were washed with PBS and up to 2x10^5^ events were acquired on a FACSCanto (BD). Results were analyzed using FlowJo 10.5.3 software. Single color controls were included in every experiment for compensation.

**FIGURE 1:**
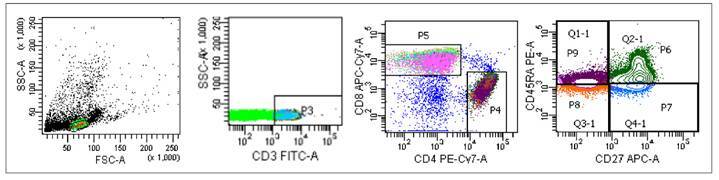
Gating for flow cytometry analysis of PBMCs (T cells subsets) from CRC patients. Gate on lymphocytes; gate on CD3 **T lymphocytes** (P3); gate on **CD4** (P4) and **CD8** (P5) T cells; from CD4^+^gate on CD45RA^+^CD27^+^ (**naпve**) (P6); gate on CD45RA^-^CD27^+^ (**Central Memory CM**) (P7); gate on CD45RA^-^CD27^-^ (**effector memory EM**) (P8); gate on CD45RA^+^CD27^-^ (**effector memory RA EMRA**).

Subsets of T cells CD4+ and CD8+ [naïve, central memory (CM), effector memory (EM), effector me mory re-expressing CD45RA (EMRA)] (Figure 1) and subsets of B cells [B naïve, B IgM memory, B switched memory, B double negative (DN)] were evaluated.

### Statistics

Results are presented as mean ± SEM. Statistical analysis was performed by using unpaired Student’s-tests and *P*≤0.05 was considered as statistically significant.

## RESULTS

Ten men and 14 women were included in this study, comprising 8 men and 4 women with stage I/II and two men and 10 women with stage III disease (Table 1). CRC patients diagnosed at stage I/II or stage III presented no significant difference in age at diagnosis, current age and months of follow-up. More cases of CRC at stage I/II were observed in men (n=8) whereas more cases of CRC at stage III were found in women (n=10). The majority of patients presented comorbidities and only one patient in stage I/II and three patients in stage III displayed no comorbidity. Our studied population is very heterogeneous clinically and patients with the same stage of disease required different therapy. Therefore, the evaluation of the immune system could help in predicting prognosis and identify those patients that could benefit from adjuvant therapies.

**TABLE 1:** Demographics of CRC patients.

Patients characteristics	CRC stage I/II (n=12)	CRC stage III (n=12)	*P*
Age at diagnosis (years)	60.2 (50-67)	61.3 (50-78)	NS
Age at 2024 (years)	66.1 (56-71)	66.3 (55-84)	NS
Follow-up (months)	60 (24-156)	37.5 (24-156)	NS
Male	8	2	
Female	4	10	
No Chemotherapy/radiotherapy	6	0	
Chemotherapy	4	8	
Chemotherapy + Radiotherapy	2	4	
Hypertension	8	6	
Diabetes mellitus	4	2	
Dyslipidemia	5	5	
Hypothyroidism	3	1	
No comorbidity	1	3	

Our initial analysis aimed to identify differences on T and B cells based on the severity of the CRC (stage I/II versus stage III). We found that there was no difference between the mean percentage of T or B cells and respective subsets when CRC patients in stage I/II were compared with CRC patients in stage III (Table 2), probably due to the patients’ heterogeneity.

**TABLE 2:** Percentage of T and B cells and respective subsets in CRC patients stage I/II compared to CRC patients stage III.

T cell subsets	CRC stage I/II	CRC stage III	*P*
**CD3%**	74.95	67.85	NS
**CD4%**	59.3	57.95	NS
Naive%	39.2	39.9	NS
Central Memory%	39.7	35.2	NS
Effector Memory%	16.1	10.6	NS
Effector Memory RA%	0.5	1.3	NS
**CD8%**	29.0	32.6	NS
Naive%	33.1	36.7	NS
Central Memory %	19.2	31.5	NS
Effector Memory %	9.0	5.2	NS
Effector Memory RA%	7.4	11.3	NS
**CD4/CD8**	2.2	1.8	NS
B cell subsets	CRC stage I/II	CRC stage III	P
**CD19%**	4.8	6.2	NS
Naive%	16.8	12.0	NS
IgM memory%	56.8	41.6	NS
Switched memory%	15.0	14.7	NS
Double negative%	14.1	14.5	NS

The results on T and B cells led us to the hypothesis that the tumor burden in association with the comorbidities of the patients (Table 1) had a potential impact in the immune system, masking possible differences displayed by T and B cells in less severe (stage I/II) or more severe (stage III) CRC cases. However, two patients progressed with disease relapse and in their registry there were no comorbidities. These patients had also in common a high percentage of double negative B cells (B DN).

Based on the mean percentage of DN B cells (14.1% - stage I/II and 14.5% - stage III, Table 1), we built the hypothesis that B DN in higher percentages could correlate with poor outcome in CRC. Considering that the mean of B DN was similar in CRC stage I/II and CRC stage III, we then divided patients in B DN lower (CRC 1) and higher (CRC 2) than 14% for the next set of analysis.

**FIGURE 2:**
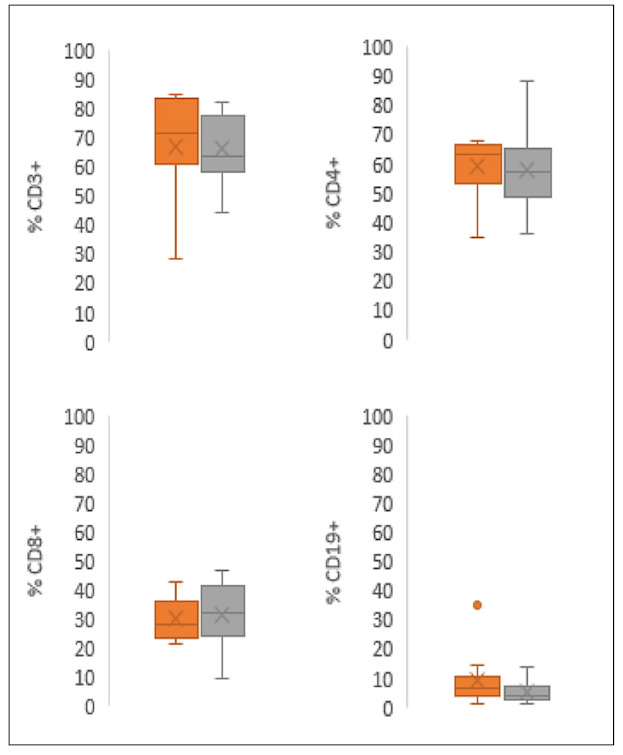
Percentage of total T cells (CD3+), T helper cells (CD4+), T cytotoxic cells (CD8+), and total B cells (CD19+) in CRC 1 (orange, B DN lower than 14%) and CRC 2 (grey, B DN higher than 14%) groups.

There was no significant difference regarding to the percentage of total CD3+, CD4+, CD8+, and CD19+ cells when CRC 1 and CRC 2 groups were compared (Figure 2).

**FIGURE 3:**
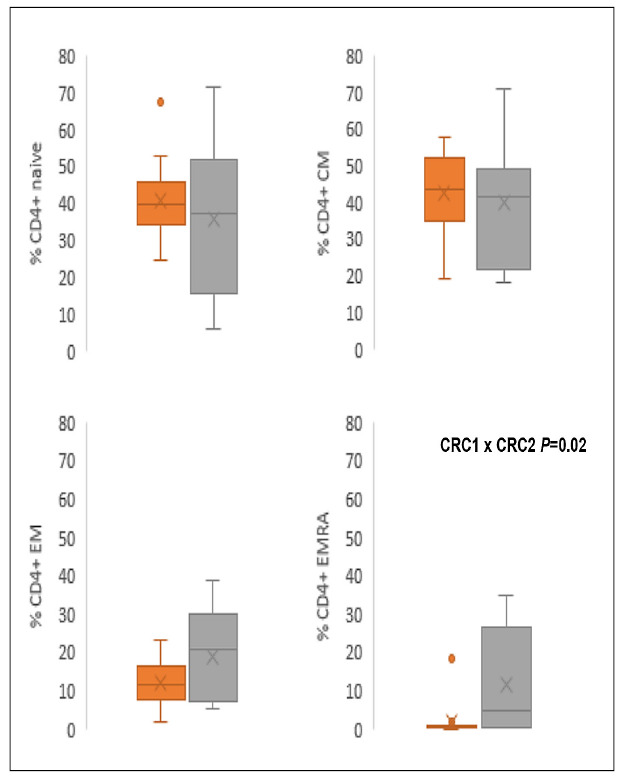
Percentage of CD4+ T cells subsets: naïve, central memory (CM), effector memory (EM) and effector memory re-expressing CD45RA (EMRA) in CRC 1 (orange, B DN lower than 14%) and CRC 2 (grey, B DN higher than 14%) groups.

The percentage of EMRA CD4+ T cells was significantly higher in CRC 2 group in comparison with CRC 1 group (Figure 3).

**FIGURE 4:**
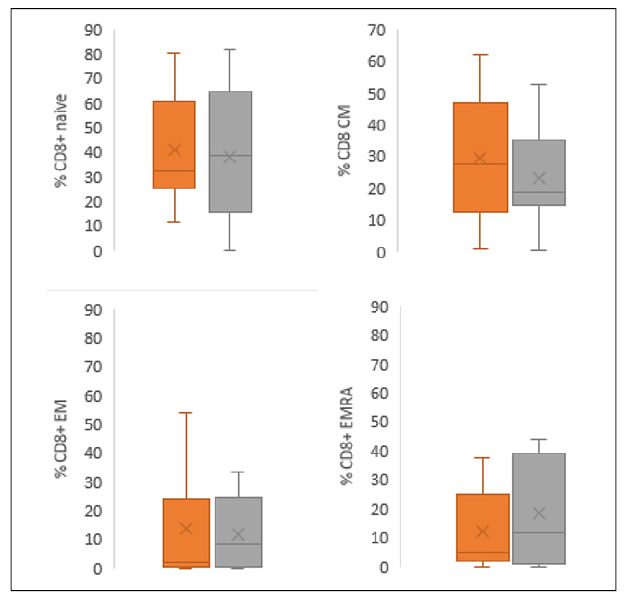
Percentage of CD8+ T cells subsets: naïve, central memory (CM), effector memory (EM) and effector memory re-expressing CD45RA (EMRA) in CRC 1 (orange, B DN lower than 14%) and CRC 2 (grey, B DN higher than 14%) groups.

There was no difference between groups CRC 1 and CRC 2 regarding to percentage of CD8+ naïve, CM, EM and EMRA T cells (Figure 4).

**FIGURE 5:**
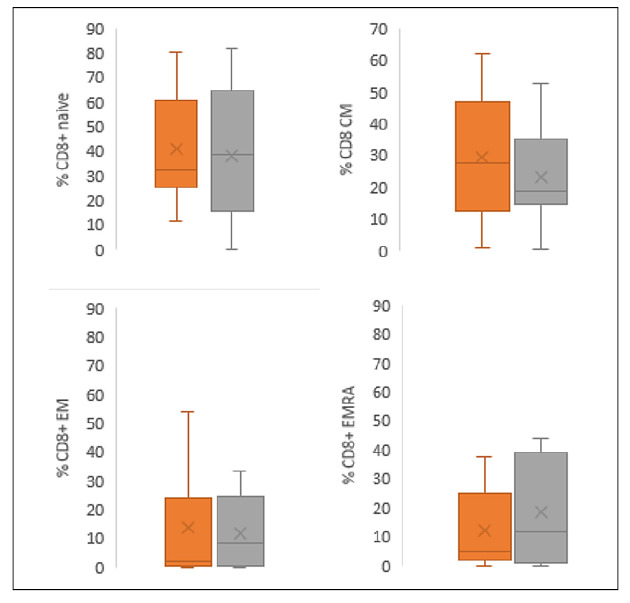
Gating strategy for flow cytometry analysis of PBMCs (B cells subsets) from CRC patients. Gate on lymphocytes; CD19 **B lymphocytes** (P3); gate on IgD^+^CD27^+^ (P4) and IgM^+^ (P5) **IgM memory**; gate on IgD^+^CD27^-^ **Naпve** (P6); gate on IgD^-^CD27^+^ **switched memory** (P8); gate on IgD^-^CD27^-^ **double negative DN** (P10).

**FIGURE 6:**
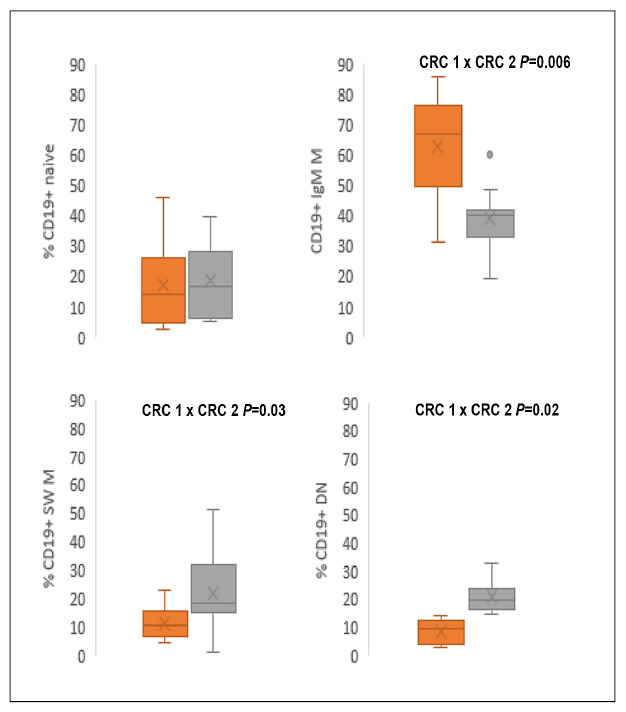
Percentage of CD19+ B cells naïve, IgM Memory (IgM M), switched memory (SW M) and double negative (DN) in CRC 1 (orange, B DN lower than 14%) and CRC 2 (grey, B DN higher than 14%) groups.

CRC 1 patients presented a higher percentage of IgM memory cells than CRC 2 patients. Patients CRC 2 presented higher percentage of switched memory and double negative B cells when compared to CRC 1 patients (Figure 5 and 6).

## DISCUSSION

Infiltration of tumor microenvironment (TME) by T cells has been correlated with better prognosis in CRC^15^ ^-^ ^17^ and also in other tumors^18^ ^,^ ^19^. B cells have only recently reached attention and, thus, articles about B cells in CRC are scarce. Another important aspect is that the investigation only in TME is not enough for the complete understanding of the anti-tumor role played by immunity. The immune system at the periphery is crucial for the body’s immune homeostasis and it is disturbed by tumor and anti-tumor therapies^20^. Ke et al. found that CD8+ T effector memory cells (TEM) in TME had a positive correlation with its counterpart in the circulating blood in CRC patients^21^. In addition, blood analysis is more suitable for evaluations in different periods during the follow-up of patients after treatment.

In the present study, CRC patients already treated and in follow-up for at least two years presented no significant difference for all evaluated subsets of T and B cells when more severe cases of CRC (stage III) were compared with less severe disease (stages I/II). In a second set of analysis we compared CRC patients displaying lower percentages of B DN cells (CRC 1) with CRC patients expressing higher percentages of B DN cells (CRC 2). We observed higher percentages of circulating CD4+ EMRA T cells in CRC 2 than in CRC 1 patients. In CRC 2 group there were 2 patients with disease relapse after 31 and 54 months of follow-up. Leon-Ferre et al. found that at pre-treatment (neoadjuvant chemotherapy) of operable breast cancer, higher percentages of CD8+ naïve and CD4+ EMRA T cells in peripheral blood were associated with minimal or no residual disease^22^. In our study, after treatment, patients of both groups (CRC 1 and CRC 2) presented no statistical difference in naïve CD8+ T cells (≥30%) whereas CD4+ EMRA T cells were present in higher percentage in CRC 2 group.

B cells have recently reached attention and Xia et al. found that the baseline percentages of IgM memory B cells in peripheral blood were higher in patients that respond to anti-PD-1 monotherapy in advanced non-small cell lung cancer (NSCLC) than in non-responders. Moreover, higher percentages of IgM memory B cells at baseline were associated with a longer progression-free survival^23^. In the present study, CRC patients with disease relapse (n=2) presented the highest percentages of B DN. Considering the whole group (CRC 2, n=11) it was also found higher percentages of switched memory B cells and lower IgM memory B cells. Regarding to switched memory B cells in prostate cancer (PCa), Saudi et al. found no statistical difference in these cells from peripheral blood when patients with PSA ≥ or ≤20 were compared. In tumor-draining and non-draining lymph nodes the percentage of B switched memory cells were similar, but the percentage of these cells in lymph nodes were significantly higher than in blood^24^. In esophageal squamous cell carcinoma, it was found that B double negative cells (B DN) were the main subset in lympho-myeloid aggregates in tumor. This finding was associated with reduced overall survival time^25^. In non-small cell lung cancer, the use of hypofractionated stereostatic radiation therapy was associated in peripheral blood (3 weeks after therapy) with increase of CD4+ and CD8+ T cells expressing TNF-alpha, IFN-gama, IL-2+, and Granzyme B+ in CD8+ T. In addition, it was observed decreased expression of naïve B cells and DN B cells whereas switched memory B cells did not change^26^. In a group of 116 kidney transplanted recipients (65 developed cancer) with present or past cancer, it was found that B switched memory cells had a significant association with cancer development^27^. Guo et al. evaluating patients with colorectal cancer and using gene expression profile, found that a longer overall survival was correlated with the abundance of class-switched memory B-cells^28^.

Our study have some limitations since although some differences had been observed based on DN B-cell values in the CR2 compared to the CR1, the number of patients that relapsed was very small. In addition, the identification of additional biomarkers and products of DN B-cells that could link them to functional activation (CD38, T-bet, CD11c, TNF-alpha, IFN-gamma) is mandatory to use these cells as prognostic biomarkers. Our ongoing studies show an increase in TNF-alpha production by B cells stimulated in vitro with PHA (unpublished data), suggesting an inflammatory profile.

In conclusion, lower IgM memory B cells and higher B DN in patients with colorectal cancer recurrence suggest a possible exploratory association between alterations in B-cell subsets and clinical outcomes; however, they fail to demonstrate validated prognostic value.

## Data Availability

Data in article: the research data are presented within the article itself (available in Tables 1 and 2).
